# PN Tandem Solar Cells Based on Combination of Dye-Sensitized TiO_2_ Photoanode and Perovskite-Sensitized NiO Photocathode

**DOI:** 10.3390/mi17010099

**Published:** 2026-01-12

**Authors:** Huan Wang, Weicheng Tang, Mengru Li, Xiaoli Mao

**Affiliations:** 1 School of Electrical Engineering and Automation, Hefei University of Technology, Hefei 230009, China; 2 School of Physics, Hefei University of Technology, Hefei 230009, China

**Keywords:** PN tandem solar cells, dye-sensitized TiO_2_ photoanode, perovskite-sensitized NiO photocathode

## Abstract

Dye-sensitized solar cells (DSSCs) have attracted significant attention as next-generation photovoltaic devices due to their low cost, simple fabrication process, use of earth-abundant materials, and potential for colour tunability and transparency. p–n tandem DSSCs have garnered particular interest owing to their higher open-circuit voltage compared to single-junction DSSCs. However, the performance of such tandem devices remains limited by relatively low open-circuit voltage and short-circuit current density, primarily due to the scarcity of suitable p-type sensitizers. To address this challenge, we report a novel p–n tandem solar cell integrating a dye-sensitized TiO_2_ photoanode with a perovskite-sensitized NiO photocathode, achieving a record power conversion efficiency of 4.02%. By optimizing the thickness of the TiO_2_ layer, a maximum open-circuit voltage of 1060 mV and a peak short-circuit current density of 6.11 mA cm^−2^ were simultaneously attained.

## 1. Introduction

Dye-sensitized solar cells (DSCs) are attractive as next-generation solar cells because of many advantages, e.g., low cost and facile fabrication, earth-abundant raw materials, colourful and transparent [1,2,3]. The theoretic upper limit of photon-to-current conversion efficiency (PCE) of DSSC is 33% [4]. However, the reported highest efficiency of DSSC is only in the range of 12–15% [5,6,7,8,9]. One of the main limiting factors is due to the light absorption range of dyes covering only within 700–800 nm, which is much narrower than that of commercialized crystalline silicon-based solar cells and other kinds of inorganic thin film solar cells. PN tandem solar cells can not only widen the absorption spectrum but also avoid adverse interactions between two kind dyes positioned separately. However, several studies have been dedicated to PN tandem quantum dot-sensitized solar cells, but have not obtained competitive performance [10,11,12].

It is evident that the current reported performance of p–n tandem dye-sensitized solar cells (DSSCs) significantly lags behind that of conventional n-type DSSCs, primarily due to the limited efficiency of p-type DSSCs. With respect to the p-type semiconductor—exemplified by NiO—an ideal sensitizer must fulfill two key criteria. First, its highest occupied molecular orbital (HOMO) level should be lower in energy than the valence band of NiO (−5.1 eV versus vacuum), while its lowest unoccupied molecular orbital (LUMO) level should be higher than the redox potential of the I_3_^−^/I^−^ couple (−4.85 eV versus vacuum). This energetic alignment ensures sufficient driving force for efficient hole injection into the valence band of NiO and simultaneous electron transfer to the electrolyte for the reduction of I_3_^−^ to I^−^. Second, the width of the absorption spectrum for the sensitizer is of great significance. The ideal sensitizer should possess broad spectral absorption across the solar spectrum to capture more photons to enable a higher short-circuit current density (*Jsc*).

It is obvious that the currently reported performance of pn tandem DSSCs is far behind that of conventional n-type DSSCs, which is limited by the poor performance of p-type DSSCs [13,14,15,16]. To date, numerous scientific researchers have dedicated significant efforts toward optimizing the performance of dye-sensitized solar cells (DSSCs). The PMI-6TTPA-TPA dye has attracted considerable attention due to its relatively broad absorption spectrum and high extinction coefficient [17,18]. P-type DSSCs based on this dye have achieved a power conversion efficiency (PCE) of 1.3%. To further enhance device performance, in 2015, Bach’s group successfully introduced the tris(acetylacetonato)iron (III)/(II) redox couple ([Fe(acac)_3_]^0^/^1−^) into p-type DSSCs, achieving an improved PCE of 2.51% [19]. Despite the passage of over a decade, the PCE of p-type DSSCs has remained largely stagnant at this level. The majority of reported devices exhibit a PCE below 1%, significantly lagging behind their n-type counterparts. This performance gap is primarily attributed to the lack of an efficient p-type sensitizer.

Organic–inorganic hybrid perovskites have attracted significant research interest due to their high extinction coefficient, bipolar charge transport characteristics, broad absorption spectrum, low material cost, and facile fabrication process. These advantageous properties have led to their widespread application in optoelectronic devices, particularly in solar cells and photodetectors [20,21,22,23]. Building upon these merits, our group previously reported an efficient CH_3_NH_3_PbI_3_-sensitized p-type mesoporous NiO solar cell employing an iodine-based liquid electrolyte, achieving a high open-circuit voltage (*Voc*) of 205 mV and a remarkable short-circuit current density (*Jsc*) of 9.47 mA cm^−2^ [24]. In this work, we successfully designed a novel pn tandem dye-sensitized solar cell by integrating an organic dye (N719)-sensitized n-type TiO_2_ photoanode and an organometal halide perovskite (CH_3_NH_3_PbI_3−x_Cl_x_)-sensitized p-type NiO photocathode, which share a common iodine-based liquid electrolyte to form a sandwich-structured device. By optimizing the thickness of the TiO_2_ layer, a record power conversion efficiency of 4.02% was achieved, along with a *Voc* of 1060 mV, a *Jsc* of 6.11 mA cm^−2^, and a fill factor (FF) of 0.62.

## 2. Experimental Section

### 2.1. Fabrication of the Perovskite-Sensitized NiO and Al_2_O_3_ Photocathode

All chemicals were of analytical grade and used as received unless otherwise specified. A compact NiO thin film was deposited on FTO glass (8 Ω sq^−1^, Nippon Sheet Glass, Tokyo, Japan) via spray pyrolysis using a 0.2 mol L^−1^ solution of nickel acetylacetonate in acetonitrile at 500 °C [22]. Subsequently, mesoporous NiO and Al_2_O_3_ films were deposited onto the prepared NiO compact layer by screen printing, employing a paste consisting of commercially available NiO and Al_2_O_3_ nanoparticles (20 nm, Inframat, Manchester, CT, USA). The resulting film was then annealed at 450 °C for 30 min, followed by annealing at 550 °C for 15 min. A bright yellow CH_3_NH_3_PbI_3−x_Cl_x_ precursor solution was prepared by dissolving 1.224 g of PbCl_2_ and 2.099 g of CH_3_NH_3_I in 5 mL of DMF and stirring the mixture at 50 °C for 2 h. The perovskite solution was then spin-coated onto the mesoporous NiO/Al_2_O_3_ film at 2000 rpm for 30 s under ambient conditions with controlled humidity (<30%), followed by thermal annealing at 100 °C for 90 min until a dark grey, uniform film formed.

### 2.2. Preparation of TiO_2_ Photoanode Sensitized by N719 and Solar Cell

A dense TiO_2_ layer was deposited on FTO glass (8 Ω sq^−1^, Nippon Sheet Glass, Japan) following a previously reported method [9]. Briefly, clean FTO glass was placed on a hot plate maintained at 450 °C. A solution of 0.38 M titanium di-isopropoxide bis(acetylacetonate) (Sigma-Aldrich, St. Louis, MO, USA) in 2-propanol was atomized and sprayed onto the substrate surface, followed by annealing at 450 °C for 30 min. Subsequently, a mesoporous TiO_2_ film was fabricated by screen-printing a TiO_2_ paste onto the compact TiO_2_ layer-coated FTO glass. The resulting film was then calcined in a stepwise manner: 375 °C for 15 min, 450 °C for 15 min, and 500 °C for 30 min. After cooling to 80 °C, the TiO_2_ films were immersed in a 0.3 mM solution of N719 dye in a 1:1 (*v*/*v*) mixture of acetonitrile and tert-butyl alcohol and allowed to adsorb at room temperature for 20 h. The electrolyte, consisting of 0.5 M LiI, 0.25 M I_2_, 0.3 M tert-butylpyridine, and 0.03 M urea in ethyl acetate, was introduced into the cell using the vacuum backfilling technique.

### 2.3. Characterizations

**General material characterizations:** The film thickness was measured using a profilometer (Dektak 150, Veeco Instruments Inc., Plainview, NY, USA). UV-vis-NIR spectra of the films were recorded with a Perkin-Elmer UV/Vis spectrophotometer (Lambda 950, PerkinElmer, Shelton, CT, USA). The crystalline phases were characterized by X-ray powder diffraction (XRD) using a PANalytical X’Pert (PANalytical, Almelo, The Netherlands) PRO diffractometer equipped with Cu Kα radiation.

**Photovoltaic characterizations:** A 450 W xenon arc lamp (Oriel, model 9119, NewPort, RI, USA) equipped with an AM 1.5G filter (Oriel, model 91192) was used to provide a simulated solar irradiance of 100 mW cm^−2^. The current–voltage characteristics of the device were measured under these conditions by applying an external potential bias and recording the resulting photocurrent using a Keithley 2400 digital source meter (Keithley, Solon, OH, USA). To minimize light scattering, a 4 × 4 mm^2^ aperture mask was placed on the solar cell active area. The same data acquisition system was employed to control the incident photon-to-current efficiency (IPCE) measurements. During IPCE measurements, a white light bias at 1% of standard sunlight intensity was applied to the sample along with an AC modulation signal (10 Hz).

## 3. Results and Discussion

The efficiency of the tandem cell (*η* = 4.02%) outperforms either of the half cells (3.24% for n-type half cell and 1.78% for p-type half cell). In comparison to the best pn tandem dye-sensitized solar cell reported in 2010 by Udo Bach, the J_sc_ of our tandem cell is much higher (6.11 mA cm^−2^ versus 2.40 mA cm^−2^), which arises from the more efficient p-type perovskite sensitizer with a wider light absorption range. The overall efficiency is therefore improved by 110.48% (4.02% versus 1.91%). Such performance is superior to all of the previously reported PN tandem solar cells based on organic dyes and quantum dots (see Table 1). The primary reason for this can be attributed to the advantageous properties of perovskites: one is their broad absorption spectrum, and the other is their bipolar charge transport characteristics. Both of these properties contribute significantly to the performance of the fabricated pn tandem solar cell.

**Figure 1:**
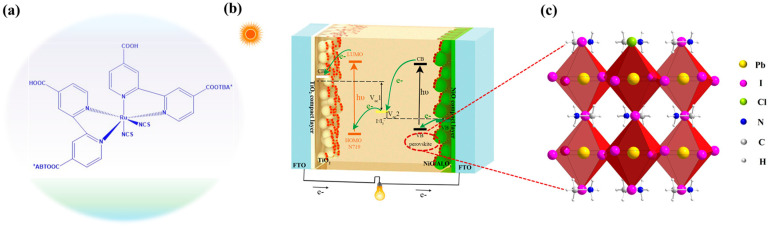
(**a**,**c**) The structures of N719 and perovskite. (**b**) Scheme representation of the tandem solar cell. The energy level (versus vacuum level, Vac) diagram of the component materials and a simple electron transfer processes are also shown.

Figure 1 represents the structure of N719 (Figure 1a) and perovskite (Figure 1b) and a schematic configuration of the pn tandem solar cell with the approximate energy levels of each component. The potential electron transfer processes within the tandem cell are also depicted. The device comprises an N719 dye-sensitized n-type TiO_2_ photoanode and an organometal halide perovskite (CH_3_NH_3_PbI_3−x_Cl_x_)-sensitized p-type NiO photocathode, arranged in a simple sandwich configuration with an intermediate electrolyte layer. Under light illumination, the N719 dye sensitizer injects electrons into the conduction band of TiO_2_ at the anode, while the CH_3_NH_3_PbI_3−x_Cl_x_ injects holes into the valence band of NiO at the cathode. The photo-oxidized N719 dye or reduced CH_3_NH_3_PbI_3−x_Cl_x_ sensitizer is then regenerated by a commonly used iodide/triiodide electrolyte. Because the two photoelectrodes are connected in series, the photovoltages of this tandem device are the sum of the corresponding p and n devices. Consequently, this tandem configuration achieves a significantly higher photovoltage and power conversion efficiency (PCE) compared to single-junction solar cells.

**Figure 2:**
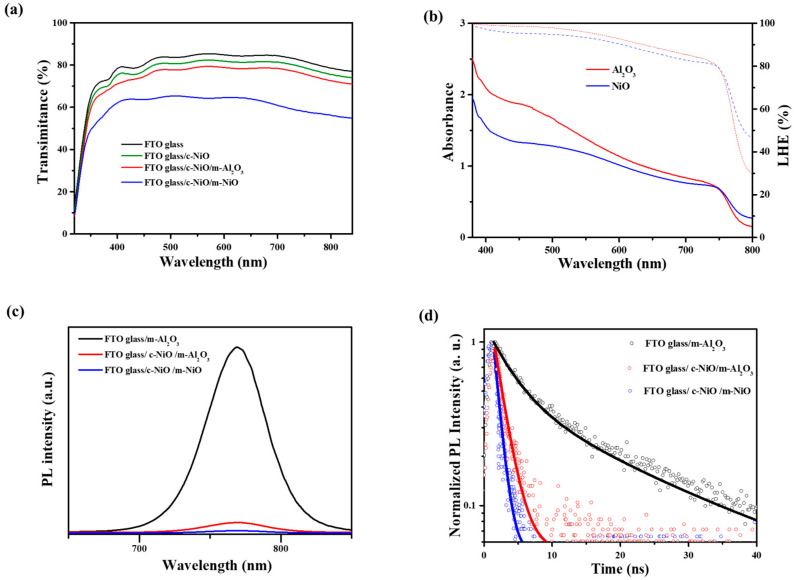
(**a**) Transmittance of FTO glass, FTO glass/c-NiO, FTO glass/c-NiO/m-Al_2_O_3_, and FTO glass/c-NiO/m-NiO. (**b**) Absorbance of pure NiO and Al_2_O_3_ films deposited with perovskite. (**c**,**d**) Steady-state and transient fluorescence spectroscopy of FTO glass/m-Al_2_O_3_, FTO glass/c-NiO/m-Al_2_O_3_, and FTO glass/c-NiO/m-NiO.

The transmittance of the different films varies significantly, as shown in Figure 2a. Notably, the FTO glass/compact NiO/mesoporous Al_2_O_3_ structure exhibits higher transmittance compared to the FTO glass/compact NiO/mesoporous NiO structure. This difference can be attributed to the larger bandgap of Al_2_O_3_ relative to that of NiO, which enhances light penetration and consequently leads to a higher photocurrent in solar cells employing the FTO glass/compact NiO/mesoporous Al_2_O_3_ configuration as photocathodes.

Furthermore, when perovskite is deposited on pure NiO and Al_2_O_3_ films, a distinct difference in absorbance is observed, as shown in Figure 2b. Compared with NiO films, Al_2_O_3_ films exhibit a higher extinction coefficient, which facilitates the capture of a greater number of photons and thereby contributes to a higher short-circuit current density in solar cell applications.

In addition, both the FTO glass/compact NiO (c-NiO)/mesoporous Al_2_O_3_ (m-Al_2_O_3_) and FTO glass/compact NiO/mesoporous NiO structures (m-NiO) were subjected to steady-state and time-resolved fluorescence measurements, as shown in Figure 2c,d. The results show that fluorescence quenching occurs rapidly in the FTO glass/compact NiO/mesoporous NiO structure, whereas the fluorescence lifetime is significantly longer in the FTO glass/compact NiO/mesoporous Al_2_O_3_ film. To accurately analyse the lifetime, we calculated the lifetimes for both samples. The carrier lifetimes are estimated to be approximately 7.8 and 3.6 ns for the FTO glass/c-NiO/m-Al_2_O_3_ and FTO glass/c-NiO/m-NiO, respectively. This indicates that porous NiO exhibits a stronger capability for capturing photogenerated carriers compared to porous Al_2_O_3_, thereby enabling more efficient hole extraction in the device and leading to a higher open-circuit voltage (Voc).

**Figure 3:**
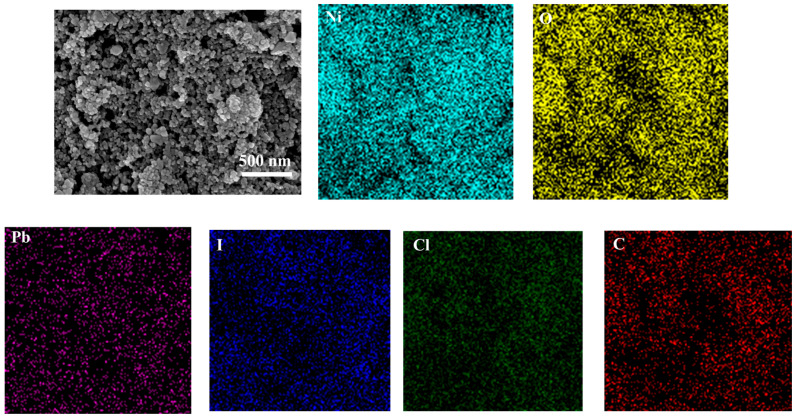
SEM of mesoporous NiO nanoparticles and EDS of Pb, I, Cl, and C elements.

To analyse the morphology of NiO and confirm the adsorption of perovskite on its surface, scanning electron microscopy (SEM) and energy-dispersive X-ray spectroscopy (EDS) were performed on the FTO/NiO substrate after perovskite deposition, as displayed in Figure 3. The results reveal that the as-prepared porous NiO film exhibits uniform particle size. EDS mapping shows a homogeneous distribution of Pb, I, Cl, and C elements across the surface, confirming the successful adsorption of perovskite onto the porous NiO layer.

**Figure 4:**
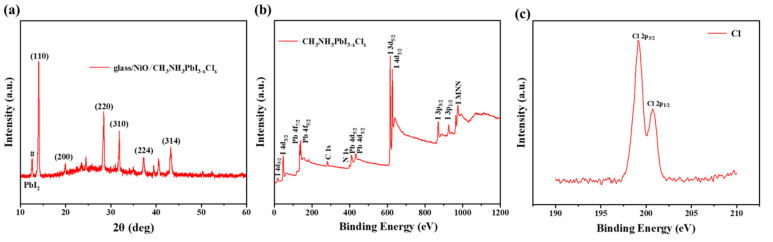
(**a**) XRD patterns and (**b**) XPS characterization of CH_3_NH_3_PbI_3−x_Cl_x_. (**c**) Cl 2p core-level XPS spectra of the perovskite films showing the characteristic doublet peaks of Cl 2p 3/2 and Cl 2p 1/2.

To analyse the crystal structure of the perovskite, X-ray diffraction (XRD) measurements were conducted, as shown in Figure 4a. The annealed MAPbI_3−x_Cl_x_ film exhibits characteristic diffraction peaks at 2θ angles of 14.08°, 28.41°, 31.85°, 40.46°, and 43.16°, corresponding to the (110), (220), (310), (224), and (314) crystal planes of the tetragonal perovskite structure, respectively, which is consistent with the previously reported literature [28]. In addition, the peak located at 12.7 degrees is attributed to PbI_2_ and is marked with a “#” symbol.To clarify the presence and chemical states of the elements in the prepared MAPbI_3−x_Cl_x_ film, X-ray photoelectron spectroscopy (XPS) was employed to analyse the elemental composition of the perovskite. As shown in Figure 4b, clear peaks corresponding to Pb, I, C, and N are observed. To detect the Cl element, high-resolution XPS spectra for Cl were acquired, as depicted in Figure 4c. The results confirm the presence of chlorine in the as-prepared perovskite film.

**Figure 5:**
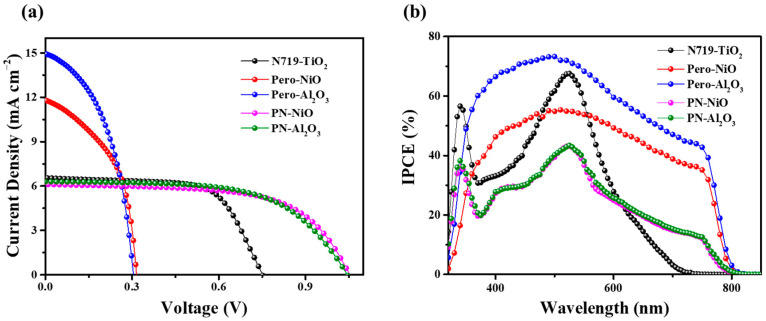
(**a**) Current–voltage characteristics and (**b**) incident photon-to-current conversion efficiency spectra of the CH_3_NH_3_PbI_3−x_Cl_x_/NiO-based p device (red), CH_3_NH_3_PbI_3−x_Cl_x_/NiO-based p device (blue), N719/TiO_2_-based n device (black), the tandem pn device based on NiO (pink), and the tandem pn device based on NiO (green). A simulated AM1.5 light (100 mW cm^−2^) was employed to illuminate from the photoanode side.

In order to fabricate a highly efficient pn tandem solar cell, we firstly have devoted effort to optimizing the performance of p- and n- single-junction solar cells. For the p part device, we have previously reported an efficient CH_3_NH_3_PbI_3_-sensitized p-type mesoporous NiO solar cell based on iodine liquid electrolyte, with a high V_oc_ of 205 mv and a remarkable J_sc_ of 9.47 mA cm^−2^. By simply replacing CH_3_NH_3_PbI_3_ with a CH_3_NH_3_PbI_3−x_Cl_x_ sensitizer, the V_oc_ of the cell was further increased to 315 mV, and J_sc_ increased to 11.80 mA cm^−2^ (as shown Figure 5 and Table 2). It is particularly necessary to point out that both the V_oc_ and J_sc_ are the highest among the p-type DSCs or p-type QDSCs based on the iodide electrolyte. In addition, a perovskite-sensitized Al_2_O_3_-based solar cell was fabricated, which achieved a short-circuit current density of 14.95 mA cm^−2^—significantly higher than that of the NiO-based device. This performance difference is primarily attributed to the intrinsic light absorption of NiO, which leads to parasitic absorption and consequently reduces the available photons for perovskite excitation, thereby lowering the short-circuit current density.

Tandem solar cells based on mesoporous Al_2_O_3_ and mesoporous NiO were fabricated, building upon the design principles of single-junction solar cells. Figure 5a shows the current voltage characteristics of a tandem cell illuminated through the photoanode. The resultant photovoltaic parameters are listed in Table 1. As clearly seen, the short-circuit current density (J_sc_ = 6.11 mA cm^−2^) of the tandem cell based on mesoporous NiO nearly equals half of the p-type solar cell (J_sc_ = 14.95 mA cm^−2^); the open-circuit voltage (*V*_oc_ = 1060 mV) nearly equals the sum of that of the n-type dye-sensitized half cell (751 mV) and p-type perovskite-sensitized half cell (315 mV); and the fill factor (FF = 0.62) is between that of the two half cells. The efficiency of the tandem cell (PCE = 4.02%) outperforms both of the half cells (3.24% for the n-type half cell and 1.78% for the p-type half cell). Although the efficiency of the dye- and perovskite-based p–n tandem sensitized solar cells exceeds that of purely dye-sensitized p–n tandem solar cells, their performance still significantly lags behind that of solid-state perovskite tandem solar cells. The primary reason is that the liquid electrolyte is inefficient in extracting and transporting charge carriers. Moreover, the solvent in the liquid electrolyte can degrade the perovskite structure.

The stacked solar cells based on Al_2_O_3_ exhibit the same pattern, but the difference is that the open-circuit voltage of the Al_2_O_3_ stacked solar cells is lower than that of the NiO stacked solar cells, while the short-circuit current density is higher than that of the NiO-based stacked solar cells. The main reason for this is the parasitic light.

The incident photon-to-electron conversion efficiencies (IPCEs) of the devices were measured as shown in Figure 5b. The dye-sensitized solar cells exhibit two distinct absorption peaks at 340 nm and 532 nm, characteristic of the N719 dye. In contrast, perovskite-sensitized Al_2_O_3_ and NiO films show broad and intense absorption across the entire spectral range from 320 to 780 nm. Notably, the absorption intensity of the perovskite-sensitized Al_2_O_3_ device is higher than that of its NiO counterpart, suggesting a greater short-circuit current density, which is consistent with the current–voltage (J–V) measurements. For the tandem solar cells based on Al_2_O_3_ and NiO, respectively, their comparable absorption intensities indicate similar short-circuit current densities.

**Figure 6:**
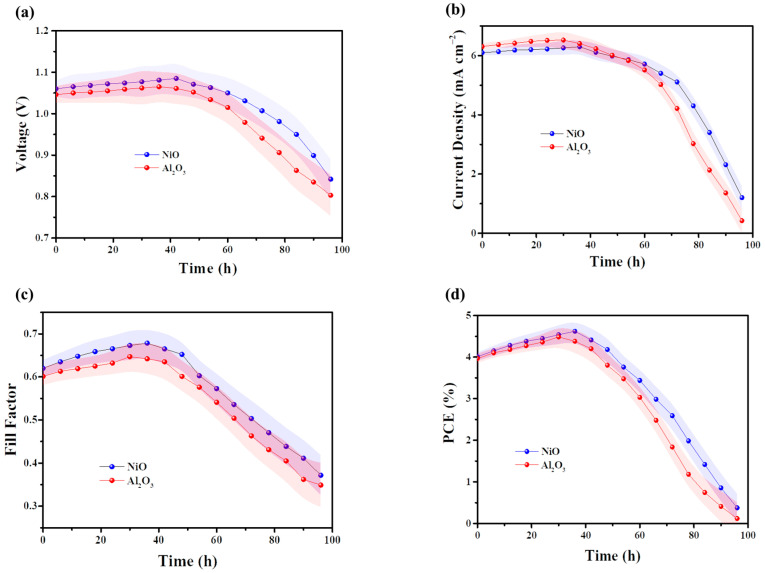
The stability of PN tandem solar cells based on NiO and Al_2_O_3_. (**a**) Voc, (**b**) Jsc, (**c**) Fill Factor, and (**d**) PCE. All of the fabricated devices were stored in a closed container at room temperature (25 ± 5 °C) and humidity (RH 25% ± 5%) and monitored periodically, with performance measurements conducted approximately every 7 h.

The stability of tandem solar cells based on NiO and Al_2_O_3_ was systematically investigated. The fabricated devices were stored in a closed container at room temperature (25 ± 5 °C) and humidity (RH 25% ± 5%) and monitored periodically, with performance measurements conducted approximately every 7 h. Both devices exhibited a slight increase in performance during the initial 40 h, followed by a rapid decline thereafter, as illustrated in Figure 6. By 100 h, the device performance had degraded to nearly zero. This pronounced degradation is primarily attributed to the use of polar organic solvents for electrolyte dissolution in this device architecture, which can readily interact with and destabilize the perovskite layer, leading to severe material decomposition and consequent performance loss.

## 4. Conclusions

In conclusion, we have for the first time demonstrated a novel double junction solar cell by using dye-sensitized porous TiO_2_ films as the photoanode and organometal halide perovskite (CH_3_NH_3_PbI_3−x_Cl_x_)-sensitized porous NiO films as the photocathode. The double junction cell achieved a photocurrent of 6.11 mA cm^−2^, a photovoltage of 1060 mV, and a record efficiency of 4.02% at 1 sun light intensity. The utilization of a perovskite-sensitized p-type semiconductor layer as an alternative to noble Pt shows great potential to convert conventional single-junction solar cells into more efficient double junction solar ones with minor changes. Compared to organic dyes, perovskite materials are cost-effective, easy to prepare, and time-efficient. Moreover, they exhibit a high extinction coefficient, bipolar charge transport characteristics, and a broad absorption spectrum. When employed as sensitizers in pn tandem-sensitized solar cells, perovskites have the potential to exceed previously reported efficiency records, including improvements in the V_oc_, J_sc_, and FF parameters. Therefore, this work opens a new path of research on highly efficient tandem-sensitized solar cells for the future.

## Figures and Tables

**Table 1:** Photoelectric properties of reported p-n tandem solar cells.

References	Cell Structure	*Voc* (mv)	*Jsc* (mA cm^−2^)	*PCE * (%)
	P + N	732	2.26	0.39%
[25]	P	83	0.269	/
	N	650	7.16	/
	P + N (N side)	918	3.62	0.66%
	P + N (P side)	887	2.73	0.78%
[26]	P	93	1.0	0.027%
	N	762	5.83	2.36%
	P + N (N side)	910	0.97	0.55%
	P + N (P side)	880	0.53	0.37%
[27]	P	350	1.66	0.2%
	N	660	1.64	0.61%
	P + N (N side)	1079	2.4	1.91%
	P	186	4.64	0.3%
	N	905	2.74	1.79%
[18]	P + N (P side)	958	4.07	2.42%
	P	227	3.87	0.3%
	N	799	11.80	5.88%

**Table 2:** Photovoltaic parameters for the pn-tandem solar cells as well as p-type solar cell and n-type DSSC.

Solar Cell Structure	*V_oc_* (mv)	*J_sc_* (mA cm^−2^)	*FF*	*PCE* (%)
p-type (NiO) solar cell	315	11.80	0.48	1.78
p-type (Al_2_O_3_) solar cell	304	14.95	0.46	2.09
n-type DSC	751	6.55	0.66	3.24
pn tandem solar cell (NiO)	1060	6.11	0.62	4.02
pn tandem solar cell (Al_2_O_3_)	1046	6.30	0.60	3.97

## Data Availability

The original contributions presented in this study are included in the article. Further inquiries can be directed to the corresponding authors.
